# A Cross-Sectional Study on the Association of Patterns and Physical Risk Factors with Musculoskeletal Disorders among Academicians in Saudi Arabia

**DOI:** 10.1155/2020/8930968

**Published:** 2020-08-15

**Authors:** Fahad Saad Algarni, Shaji John Kachanathu, Sami S. AlAbdulwahab

**Affiliations:** Department of Rehabilitation Health Sciences, College of Applied Medical Sciences, King Saud University, Riyadh, Saudi Arabia

## Abstract

**Background:**

Musculoskeletal disorders (MSDs) are considered one of the most common health issues in working population and have a high social and economic impact. This study is aimed at determining the MSD patterns and associated risk factors among higher education academicians in Saudi Arabia.

**Methods:**

A cross-sectional study was conducted among higher education academicians, randomly selected from different universities within Saudi Arabia. A sample of 207 academicians participated in the present study from different faculties such as nursing, applied medical sciences, pharmacy, dentistry, computer science, science, and engineering for a period of 1 year. The Nordic Musculoskeletal Questionnaire (NMQ-E) was used to assess the MSD patterns and prevalence for the different parts of the body regions, and the Dutch Musculoskeletal Questionnaire (DMQ) was used to determine the physical risk factors associated with the working conditions in the higher academic occupations. Descriptive statistics and the Pearson chi-squared test were used for data analysis.

**Results:**

The overall prevalence rate was 42.5%, and the pattern of body parts involved was almost similar on both study variables, i.e., descending from the lower back (31.9%), followed by the neck (26.1%), knees (21.3%), shoulder (16.9%), upper back (13%), ankle and foot (10.1%), wrist and hand (7.2%), and elbow (6.3%), and the least common observed region was the hip and thigh (2.4%). The physical risk factors and its association with the body regions based on DMQ related to workload, period of use, and repetitive movements were observed in the wrist and hand (43%), followed by the neck (42%) and trunk (21%).

**Conclusion:**

The study demonstrated that the MSDs are lower among the higher academic occupations. However, the most common MSDs observed in this group of subjects are the back, neck, and knee pain, and it is found that some of the lifetime physical activities also have a significant association with these involved body regions.

## 1. Introduction

Musculoskeletal disorders (MSDs) represent conditions that affect the muscles, tendons, ligaments, joints, cartilages, peripheral nerves, and spinal discs in the body that may be associated with exposure to risk factors in the workplace [1]. The World Health Organization (WHO) has described that multifactorial risk factors were responsible for work-related MSDs (WMSDs) among workers across the globe. MSDs account for a significant proportion of the disease burden worldwide and have considerable economic implications. It is reported to be a major problem in the working industries with back and shoulder disorders being the most common and costly disorders [2].

Research studies had been conducted on the prevalence and physical risk factors of MSDs among various occupations such as agriculture workers [3], office workers [4], school teachers [5], and health care professionals in different countries [6]. However, in academicians, the prevalence of MSDs ranged between 39% and 95% [7] varied among countries related to occupational and environmental conditions due to lack of adequate resources [8] and social and geographical factors [9]. Generally, it has been reported that the back, neck, and upper limb regions are the most frequently affected. It was observed that there was some relation between the professional categories and the involvement of body parts [10]. Moreover, studies reported that the nature of physical work in academicians such as prolonged standing and sitting and uncomfortable posture is known to be associated with an increased prevalence of MSDs [11]. Previous studies reported that single or cumulative trauma of continuous exposure to risk factors may lead to MSDs [12]. In addition to physical risk factors, it has been suggested that psychosocial factors also play a role in increasing workload demands and perceived stress levels. Low social support, occupational control, satisfaction, and being monotonous in occupations are associated with MSDs among school teachers [7].

In recent years, occupational health and physical risk management is the major concern of any organization. MSDs have become an increasing affair to employees, employers, and governments because of the impact on workers' health, labor absenteeism, and productivity. However, researchers have paid little attention in defining exactly what constitutes MSDs related to academicians. Moreover, a paucity of demographical evidence exists in working and health conditions. Furthermore, MSD prevalence and its association with physical risk factors had not been studied among academic institutions in the Middle East countries and especially in Saudi Arabia. MSDs have a substantial and detrimental effect on the individual, societal, and economic burden in all the countries and are considered a common reason for discontinuing work and seeking health care [3]. Therefore, the objective of our study was to determine the prevalence of musculoskeletal disorders on body parts and its association with physical risk factors among the Saudi Arabian academicians.

## 2. Subjects and Methods

A cross-sectional study was conducted in randomly selected academicians from various higher educational universities within Saudi Arabia. In the present study, MSDs are defined as injuries or disorders of the muscles, nerves, tendons, joints, cartilage, and spinal discs that may be associated with work environment and performance of work. They contribute significantly to the specific condition, made worse or persist longer [1]. A total of 350 paper-based questionnaires were distributed directly to participants, out of which 227 questionnaires were returned. The study was conducted between September 2018 and May 2019, and the sample size was based on nonprobability with a convenient sampling technique. However, 20 respondents were further excluded from this study, as the questionnaires were not completed as per the study criteria. The participants were recruited from different faculties: nursing (*n* = 09), applied medical sciences (*n* = 35), pharmacy (*n* = 18), dentistry (*n* = 12), computer and information science (*n* = 17), science (*n* = 38), engineering (*n* = 73), and other streams (*n* = 05). The selected participants were recruited based on full-time working academicians, aged 23 years or older with working experience of more than 12 months in the permanent position. The participants excluded were those who reported any history of fractures and soft tissue injuries in any body region in the past 12 months; had congenital spinal disorders, scoliosis, disc protrusion, spine malformation, ankylosing spondylitis, cancer, trauma, and gynecological diseases; had pain due to surgery, tumor vessel lesions, and irregular menstruation cycle; and had long-term use of analgesics and also those with a history of psychiatric disorders. The investigators met the participants in person to explain the study objectives, rationale, and process. Every participant had read and signed the consent form prior to participating in the study. The research and ethical committee of the College of Applied Medical Sciences at King Saud University had approved the present study.

### 2.1. Measurement Tools

The extended Nordic Musculoskeletal Questionnaire (NMQ-E) and the short version of the Dutch Musculoskeletal Questionnaire (DMQ) were used in this study [13, 14]. The NMQ-E was used to assess the patterns and prevalence of MSDs for the different parts of the body regions in the last 12 months. DMQ was used to determine lifetime patterns and prevalence of MSDs and the physical risk factors associated with the academic working conditions. Both scales were distributed among all participants. The data was collected at the participant's respective workplace under the supervision of the researcher. It took about 15-20 minutes to complete both questionnaires. Prior to the collection of data, a pilot testing of both questionnaires was carried out among 10 subjects and they responded well.

#### 2.1.1. Extended Nordic Musculoskeletal Questionnaire

The NMQ-E consisted of general questions on the history of having trouble in any of the nine body regions: neck, upper back, lower back, shoulder, elbow, hand/wrist, hip, knee, and ankle/foot. This questionnaire was accompanied by a body map diagram, which facilitated the subjects to locate their pain or discomfort sites in their bodies. In addition, questions were also asked regarding the subject's lifetime experiences, followed by the prevalent questions, and, lastly, on the items related to consequences of pain in the whole year. The response categories were restricted to “yes” and “no.” The NMQ-E has been shown to be reliable for collecting information about the onset, prevalence, and consequences of musculoskeletal pain in the nine body regions [13].

#### 2.1.2. Dutch Musculoskeletal Questionnaire

The Dutch Musculoskeletal Questionnaire (DMQ) is a reliable and valid self-reported tool for identifying risk factors of musculoskeletal disorders in seven different dimensions at the workplace [14]. The short version of the DMQ consists of the areas of general, health 2, work 1, and work 2 from the standard version of the DMQ. We identified 20 questions according to the job nature of the academicians from the standard version of DMQ and listed the same in work 1 of the shortened version of DMQ [15]. The scores were rated under 4 categories: never (1 point), sometimes (2 points), often (3 points), and always (4 points). The maximum score obtained was 80, and the least score was 20 in this questionnaire.

### 2.2. Data Analysis

All statistical analyses were performed using SPSS statistical software, version 25 (IBM Inc., Chicago, IL, USA). The descriptive statistics for all the participants were expressed as mean ± standard deviation (SD). The frequency and percentage were calculated for categorical variables. The prevalence of pain in each body region was carried out by cross-tabulation and the chi-squared test used to explore the risk factors among the participants. The data were normally distributed, and the *p* value was set at ≤0.05. The test for homogeneity of variance revealed that there was no significant difference with *p* values > 0.05.

## 3. Results

The number of participants who received the questionnaires was 207. All participants completed the questionnaires without missing any information. The total number of respondents included in this study was 207 with a mean age of 44.9 ± 11.5 years and experience of 11.5 ± 8.9 years. The BMI of participants was 29.5 ± 13.4. The majority of participants were right-handed. The majority of the participants were Ph.D. and MSc degree holders: 75.4% and 19.8%, respectively. The majority of academicians were from colleges of engineering, science, and applied medical sciences: 35.5%, 18.4%, and 16.9%, respectively. The monthly salary was US$3000 or more for the majority of participants (78.7%). The majority of participants had five years of experience (75.8%), and the overall working hours was 7.4 ± 1.4. The nonsmokers were the majority (81.6%), and most of the participants considered themselves as healthy (86%). The satisfaction status was very high among the academicians (95.7%).

According to the NMQ-E, the annual prevalence of MSDs among the academicians was 31.9% in the lower back, 26.1% in the neck, 21.3% in the knees, and 16.9% in the shoulders, and the rest of the body regions had prevalence between 2.4% and 13%. The lifetime prevalence of MSDs based on DMQ among academicians was 31.9% in the lower back, 22.2% in the neck, 20.3% in the knees, and 11.6% in the shoulders, and the rest of the body regions had prevalence between 2.4% and 10.1% (Table 1).

The overall prevalence of MSDs among the academicians was 42.5% (*n* = 88). The study participant's age, educational level, monthly salary, employment duration, and smoking status except perceived health status have no correlation contribution with observed MSD prevalence (*p* > 0.05) (Table 2). The contribution of lifetime physical risk factors associated with MSDs in the study participants is presented in Table 3.

## 4. Discussion

The current study observed 42.5% annual prevalence of MSDs among the academicians in Saudi Arabia. As compared to previous studies, the present study observes lesser prevalence and change in the ranking of the involvement of body regions may be due to the difference among the participation of various faculties, wide demographics, and different work settings [7, 11, 15]. A previously done study, reported a 12-month NMQ prevalence of 55% WMSDs among faculty members of only Majmaah University with the sample size of 110 [16], whereas our current study included wide ranges of academic faculties such as nursing, applied medical sciences, pharmacy, dentistry, computer science, science, and engineering from different universities from different demographical regions of Saudi Arabia with larger sample size. The differences in the prevalence of MSDs between various countries and regions may be due to the difference of working conditions. Faculty-student ratios in various departments may also result in differences in their workloads. However, these discrepancies may explain the existing difference in the prevalence of MSDs in academicians employed in different regions. Changes in disease estimates between countries or overtime may be due to shifts in the epidemiological profile driven by socioeconomic changes, which contribute to the rate of increase in years lived with disabilities [17].

It has been observed that almost a similar prevalence pattern of MSDs occurs on body parts on both the annual NMQ-E and lifetime DMQ scores. We found that the lower back (31.9%) was the most prevalent body region, followed by the neck (26.1%), knees (21.3%), shoulder (16.9%), upper back (13%), ankle and foot (10.1%), wrist and hand (7.2%), and elbow (6.3%), and the least common region was the hip and thigh (2.4%). The current result of NMQ and DMQ prevalence similarities emphasizes the importance of frequent NMQ assessment of MSDs to prevent lifetime disabilities and help an individual lead a quality life. It has been reported that NMQ assessment is the most commonly used base instrument to assess the MSDs [15]. Interestingly, global trends report that lower back pain and neck pain are the single largest cause with little change in their rates for the past many years; moreover, rates of osteoarthritis increased recently [18]. These reviews are also coinciding with our study observations. Our study is consistent with the recent observations on work-related MSDs that the most commonly affected body regions were the lower back, neck, and shoulder [15, 19], and furthermore, back disorders are considered among the most commonly affected regions in any working industry [2]. The occupational and demographic factors also influence the prevalence of MSD patterns [11, 19].

Moreover, it is also observed that some of the lifetime physical activities of the participants such as bending or twisting movements with the trunk, neck, and wrist and hand; repetitive movements with the trunk, hands, and fingers; bending, stooping, or twisting posture for long periods; holding arms at or above shoulder level; and prolonged working periods in the same posture have a significant association with MSDs. It has been observed that the MSDs among academicians can be attributed to extrinsic factors that include the physical demands of the job like repetitive activities of bending and twisting of the trunk, neck, and wrist and hand during demonstration classes. Moreover, prolonged use of body regions in a particular posture such as standing especially during lectures attributed to MSDs in the trunk, neck, and knees [20]. Academicians are exposed to ergonomic risk factors including prolonged standing, prolonged sitting, working with computers, and walking up and down the stairs, and working with loads had a higher prevalence of MSDs [11, 15]. The use of electronic devices and computers in academics has increased dramatically in the past two decades as compared to traditional blackboard teaching. In relation to technology adaptation, previous research studies have shown that most computer users are likely to experience MSDs [11]. Ergonomic risk factors such as workstation design, awkward posture, repetitive movements, static postures, and long working hours are linked to MSDs [16].

Studies have observed that the working experiences of ≥6 years and duration of >40 hours per week were considered associated risk factors for the MSDs [19]. In the current study, the study participants had work experience of 11.5 ± 8.9 years and working time was in a range of 30-50 hours within the university campus along with other academic-related activities outside the campus. Our study showed that the prevalence of MSDs is not dependent on the body weight and working hours; this is consistent with the previous report that there is no correlation of the number of working hours per day and BMI with the prevalence of pain. The literature has shown that there is an exposure-response relationship correlating the prevalence of MSDs to the intensity, frequency, or duration of an exposure [20].

However, the study showed that the participant's age, educational level, monthly salary, employment duration, and smoking status have no correlative contribution with observed MSD prevalence. Review studies revealed a range of different risk factors for MSDs in different categories. Furthermore, in studies among academicians, sociodemographic, occupational, and other risk factors did not show any similarities [12, 20]. Furthermore, longer exposure to ergonomic risk factors at work was not reported by the previous studies [5, 11, 21]. A recent systematic review reported that the prevalence and pattern of MSDs of cooccurring back pain and its association with age, sex, or back-related disability had not been studied extensively [22]. All our study participants were males; we did not have access to study the role of MSDs in the female academic section. Therefore, we recommend studies in the future to understand the prevalence and patterns of MSD in female academicians and its association with other descriptive factors. This would support the higher academic institution's researchers and policymakers in identifying priorities.

Our study participants mostly included male academicians with higher education levels which could be another factor predisposing for MSDs. This is supported by previous observation that men had higher prevalence of stress compared to women whereas the lower education level was associated with lower stress [21]. Occupational demands and career development ambitions among educators were positively associated with stress [21, 23].

In conclusion, it appears that there is a lack of general review of health conditions among higher educators in Saudi Arabia with the recent MSD prevalence among academicians and identifying physical risk factors associated with insight and opportunity for relevant authorities, specifically the Ministry of Education to address the ergonomic problems among educators through policymaking. The present study result emphasizes the role of MSD awareness, ergonomic training, and exercise among Saudi Arabian academicians.

## Figures and Tables

**Table 1:** The annual NMQ-E and lifetime DMQ prevalence of MSDs (ache, pain, discomfort, and numbness).

Body region	NMQ-E	DMQ	Frequency	NMQ-E (%)	DMQ (%)
Neck	54	46	Yes	26.1	22.2
	153	161	No	73.9	77.8
Shoulders	35	24	Yes	16.9	11.6
	172	183	No	83.1	88.4
Upper back	27	21	Yes	13.0	10.1
	180	186	No	87.0	89.9
Elbows	13	9	Yes	6.3	4.3
	194	198	No	93.7	95.7
Wrists/hands	15	9	Yes	7.2	4.3
	192	198	No	92.8	95.7
Lower back	66	66	Yes	31.9	31.9
	141	141	No	68.1	68.1
Hips/thighs	5	5	Yes	2.4	2.4
	202	202	No	97.6	97.6
Knees	44	42	Yes	21.3	20.3
	163	165	No	78.7	79.7
Ankles/feet	21	17	Yes	10.1	8.2
	186	190	No	89.9	91.8
	207	207	Total	100.0	100.0

**Table 2:** Annual descriptive data association with MSDs.

Variables		MSDs (1-year prevalence)				
		F	No	Yes	Total	*p* value
Overall prevalence	Category	*N*	119	88	207	
		%	57.5	42.5	100	
Age	≤33	*N*	18	21	39	0.34
		%	0.46	0.54	1.00	
	34-43	*N*	27	38	65	
		%	0.42	0.58	1.00	
	44-53	*N*	15	35	50	
		%	0.30	0.70	1.00	
	54-63	*N*	15	24	39	
		%	0.38	0.62	1.00	
	≥64	*N*	8	6	14	
		%	0.57	0.43	1.00	
		%	0.4	0.6	1.0	
Education	Ph.D.	*N*	60.0	96.0	156.0	0.61
		%	0.4	0.6	1.0	
	MSc	*N*	18.0	23.0	41.0	
		%	0.4	0.6	1.0	
	BSc	*N*	2.0	4.0	6.0	
		%	0.3	0.7	1.0	
	Other	*N*	3.0	1.0	4.0	
		%	0.8	0.3	1.0	
Monthly salary	>USD$3000	*N*	63.0	100.0	163.0	0.41
		%	0.4	0.6	1.0	
	≤USD$3000	*N*	20.0	24.0	44.0	
		%	0.5	0.5	1.0	
Employment duration	1 to 5	*N*	23.0	26.0	49.0	0.26
		%	0.5	0.5	1.0	
	>5	*N*	60.0	98.0	158.0	
		%	0.4	0.6	1.0	
Do you consider yourself healthy?	No	*N*	4.0	25.0	29.0	0.0001^∗^
		%	0.1	0.9	1.0	
	Yes	*N*	79.0	99.0	178.0	
		%	0.4	0.6	1.0	
Smoking status	Current smoker	*N*	7.0	15.0	22.0	0.69
		%	0.3	0.7	1.0	
	Nonsmoker	*N*	69.0	100.0	169.0	
		%	0.4	0.6	1.0	
	Previous smoker	*N*	7.0	9.0	16.0	
		%	0.4	0.6	1.0	

**Table 3:** Association of physical risk factors and MSD based on DMQ.

Physical risk factors (lifetime)	Yes (%)	No (%)	*χ* ^2^	*p* value
Lift, pull, push, or carry loads (exceeding 20 kg)	9.0 (4.3)	198.0 (95.7)	3.29	0.09
Exert great force on tools	10.0 (4.8)	197.0 (95.2)	0.00	1.00
Bend or twist with the trunk	45.0 (21.7)	162.0 (78.3)	7.65	0.01^∗^
Bend or twist with the neck	89.0 (43.0)	118.0 (57.0)	4.85	0.03^∗^
Bend or twist with the wrists/hands	91.0 (44.0)	116.0 (56.0)	4.58	0.03^∗^
Bent, stooped, or twisted posture for long periods with the trunk	40.0 (19.3)	167.0 (80.7)	8.34	0.0001^∗^
Bent, stooped, or twisted posture for long periods with the neck	62.0 (30.0)	145.0 (70.0)	4.51	0.03^∗^
Bent, stooped, or twisted posture for long periods with the wrists/hands	68.0 (32.9)	139.0 (67.1)	2.53	0.11
Short repetitive movements with the trunk	40.0 (19.3)	167.0 (80.7)	4.71	0.03
Short repetitive movements with the neck	69.0 (33.3)	138.0 (66.7)	0.25	0.62
Short repetitive movements with the wrists	108.0 (52.2)	99.0 (47.8)	1.49	0.22
Bent, stooped, or twisted posture for long periods, reach with the arms or hands	96.0 (46.4)	111.0 (53.6)	2.44	0.12
Bent, stooped, or twisted posture for long periods, hold your arms at or above shoulder level	60.0 (29.0)	147.0 (71.0)	4.87	0.03^∗^
Working prolonged periods in the same posture	131.0 (63.3)	76.0 (36.7)	9.59	0.0001^∗^
Working prolonged periods in uncomfortable postures	65.0 (31.4)	142.0 (68.6)	3.43	0.06
Frequent repetitive movements with the arms, hands, or fingers	116.0 (56.0)	91.0 (44.0)	7.39	0.01^∗^
Standing for long periods	137.0 (66.2)	70.0 (33.8)	0.34	0.56
Sitting for long periods	146.0 (70.5)	61.0 (29.5)	2.00	0.16
Walking for long periods	54.0 (26.1)	153.0 (73.9)	1.17	0.28
Long periods of time have to kneel or squat	14.0 (6.8)	193.0 (93.2)	0.83	0.36
Slipping or falling some times during task	23.0 (11.1)	184.0 (88.9)	1.01	0.32
Enough space to complete task	188.0 (90.8)	19.0 (9.2)	0.09	0.76
Often hold vibrating tools or materials	21.0 (10.1)	186.0 (89.9)	1.29	0.26

^∗^Level of significance, *p* < 0.05.

## Data Availability

The study data used to support the findings of this study are available from the corresponding author upon request.
